# Polymorphism and Red Photoluminescence Emission from 5s^2^ Electron Pairs of Sb(III) in a New One-Dimensional Organic–Inorganic Hybrid Based on Methylhydrazine: MHy_2_SbI_5_

**DOI:** 10.3390/molecules29020455

**Published:** 2024-01-17

**Authors:** Magdalena Rowińska, Dagmara Stefańska, Tamara J. Bednarchuk, Jan K. Zaręba, Ryszard Jakubas, Anna Gągor

**Affiliations:** 1 Institute of Low Temperature and Structure Research, Polish Academy of Sciences, Okólna 2, 50-422 Wrocław, Polandt.bednarchuk@intibs.pl (T.J.B.); 2 Advanced Materials Engineering and Modelling Group, Wrocław University of Science and Technology, Wyb. Wyspiańskiego 27, 50-370 Wrocław, Poland; 3 Faculty of Chemistry, University of Wrocław, F. Joliot-Curie 14, 50-383 Wrocław, Poland

**Keywords:** methylhydrazine, red PL, organic–inorganic perovskite, phase transition, antimony iodide

## Abstract

We explore the crystal structure and luminescent properties of a new 1D organic–inorganic hybrid, MHy_2_SbI_5_, based on methylhydrazine. The compound reveals the red photoluminescence (PL) originating from the 5s^2^ electron pairs of Sb(III) as well as complex structural behavior. MHy_2_SbI_5_ crystalizes in two polymorphic forms (**I** and **II**) with distinct thermal properties and structural characteristics. Polymorph **I** adopts the acentric *P*2_1_2_1_2_1_ chiral space group confirmed by SHG, and, despite a thermally activated disorder of MHy, does not show any phase transitions, while polymorph **II** undergoes reversible low-temperature phase transition and high-temperature reconstructive transformation to polymorph **I**. The crystal structures of both forms consist of 1D perovskite zig-zag chains of corner-sharing SbI_6_ octahedra. The intriguing phase transition behavior of **II** is associated with the unstable arrangement of the [SbI_5_]^2−^_∞_ chains in the structure. The energy band gap (E_g_) values, estimated based on the UV-Vis absorption spectra, indicate that both polymorphs have band gaps, with E_g_ values of 2.01 eV for polymorph **I** and 2.12 eV for polymorph **II**.

## 1. Introduction

In recent years, there has been a significant surge of interest in organic–inorganic materials with the perovskite structure, driven particularly by the excellent photovoltaic parameters of methylammonium lead iodide (MAPI). The solar cells based on this organic–inorganic semiconductor have undergone a revolutionary transformation, increasing the efficiency of performance up to 25% within a short period [1,2]. This advancement was attributed to extensive chemical engineering through chemical substitutions, in both the molecular and inorganic parts, particularly involving halogen site mixing and co-doping with other organic cations [3,4,5]. In addition to their use in photovoltaics, other organic–inorganic perovskite analogues have found widespread applications in various optoelectronic devices, such as light-emitting diodes (LEDs) [6,7,8,9], photodetectors [10], and dielectric switchers [11,12]. Some of them exhibit a highly efficient multiphoton-excited photoluminescence (PL) up-conversion that makes them suitable for many important applications such as in vivo imaging or photodynamic therapy [13].

Recent reports show that lead halide perovskites and their 2D derivatives can be successfully obtained with methylhydrazinium cation (MHy) [14,15,16,17]. However, due to the larger effective ionic radius compared to other perovskites such as methylammonium (MA) or formamidinium (FA) and a large dipole moment (3.24D compared to 2.26D in MA and 0.22D in FA), the properties of MHy-based perovskites differ significantly from their analogs. Both MHyPbBr_3_ and MHyPbCl_3_ crystallize in polar *P*2_1_ symmetry, and the crystal structure is built of exceptionally distorted PbX_6_ octahedra. Both materials show strong second-harmonic generation activity, one-photon photoluminescence and two-photon up-conversion photoluminescence. MHy also acts as a spacer in 2D (layered) perovskites, where it prompts room-temperature ferroelectric and low-temperature polar long-range order in MHy_2_PbBr_4_ and MHy_2_PbCl_4_, respectively. Thus, MHy-molecular ions have high potential for symmetry breaking, which in addition to photovoltaic and light-emitting properties should also lead to attractive nonlinear optical (NLO), piezo-, pyro- and ferroelectric properties. It is worth mentioning that the smallest separation distance between the [PbI_4_]^2−^_∞_ layers in MHy_2_PbI_4_ exhibited an exceptionally small bandgap (2.20 eV) and significantly reduced the dielectric confinement. Recently, MHy was employed as a perovskite in the construction of a polar multilayer hybrid perovskite, (IBA)_2_MHy_2_Pb_3_Br_10_ [18].

This brief survey demonstrates that MHy-based 3D and 2D perovskites exhibit intriguing properties that are quite different from properties of known analogs crystallizing with small polar amines. We thus aim to extend studies on other MHy-inorganic compounds, as they are still not recognized well. It is noteworthy that the first methylhydrazinium lead halide (MHy_2_PbI_4_) was reported in 2019 [17]. Since then, MHy has proven its ability for creating strong hydrogen bonds with halide acceptors that lead to distortions of the inorganic framework and play a major role in polar ordering.

A significant drawback hindering the large-scale commercialization of lead halides is the presence of lead in their structure. Simultaneously, intensive research efforts are underway to explore alternatives that do not contain lead and have a smaller negative impact on the environment [19,20,21]. One way is the substitution for lead by other elements including Ge(II), Cu(II), Sb(III) or Bi(III), which lead to structures of lower dimensionality. They can adopt either a zero-dimensional (0D) structure containing isolated octahedra [22], (M_2_X_9_)^3–^ dimer units [23] or 1D chains [24,25,26,27,28,29]. Among these, there is particular interest in bismuth and antimony iodide-based compounds, primarily due to the potential semiconducting properties of the inorganic skeleton and the diverse range of structures exhibited by these systems, especially those characterized by acentric phases [30,31,32,33,34,35]. 

The presence of a lone 5s^2^ electron pair in the electronic configuration of Sb(III) and a lone 6s^2^ electron pair in Bi(III) can induce pronounced structural distortions, giving rise to electronic states that reside high in the valence band, shaping the electronic properties of these hybrids [36,37]. A lot of reports show that structural distortions and the dimensionality of the inorganic part are closely related to the photoluminescence of organic–inorganic halides. Weak deformations result in narrow PL bands and small Stokes shifts. Large structural distortions imply red-shifted and broad PL bands and large Stokes shifts. On the other hand, existing literature data on the key optoelectronic (exciton-binding energies, charge-carrier mobilities) and PL properties of organic–inorganic halides clearly indicate the importance of dynamics of the organic spacers in these systems [29,30]. 

The stereo-active effects in light-emitting 0D 5s^2^ lone pair materials have been summarized in [38]. The luminescent properties of Sb(III)-based halides have been reported mostly for materials with 0D [SbCl_5_]^2−^ square pyramidal units isolated by large organic cations [39,40,41], but also for octahedral or polynuclear octahedral units [42,43]. However, less active lone pairs in MX_6_ octahedra also exhibit the tendency towards off-center displacements and dynamic distortions. Luminescent MI_6_ and MBr_6_ octahedra have mainly been found for Sn(II) halides [44]. RT PL has also been reported for [Sb_2_I_9_]^3−^ dimers in Cs_3_Sb_2_I_9_, but with rather weak emission intensity [45]. 

In contrast to 0D discrete polyhedral units, the luminescent properties of 1D polymeric halides remain poorly understood, even though some of them exhibit efficient emissions [46]. This work constitutes another contribution to fill this gap. We delve into the connections between structure and properties that govern the performance of one-dimensional, emissive 5s^2^ MHy-based antimony(III) iodides with extended 1D zig-zag chains of corner-sharing SbI_6_ octahedra. Hybrids with the general formula R_2_SbI_5_ have already garnered significant attention from scientists as potential functional materials. This is owing to their outstanding properties, which include a strong second harmonic generation (SHG) response [32,47,48], ferroelectricity [48,49], a small bandgap with strong absorption ability [31,33,34,48,49,50,51,52], switchable dielectric properties [53] and excellent thermal stability [31]. Herein, we show the crystal structure and temperature behavior of two new polymorphic forms, temperature dependent structural phase transitions, thermal characteristics as well as luminescent properties. 

## 2. Results

### 2.1. Thermal Properties of MHy_2_SbI_5_

**Figure 1:**
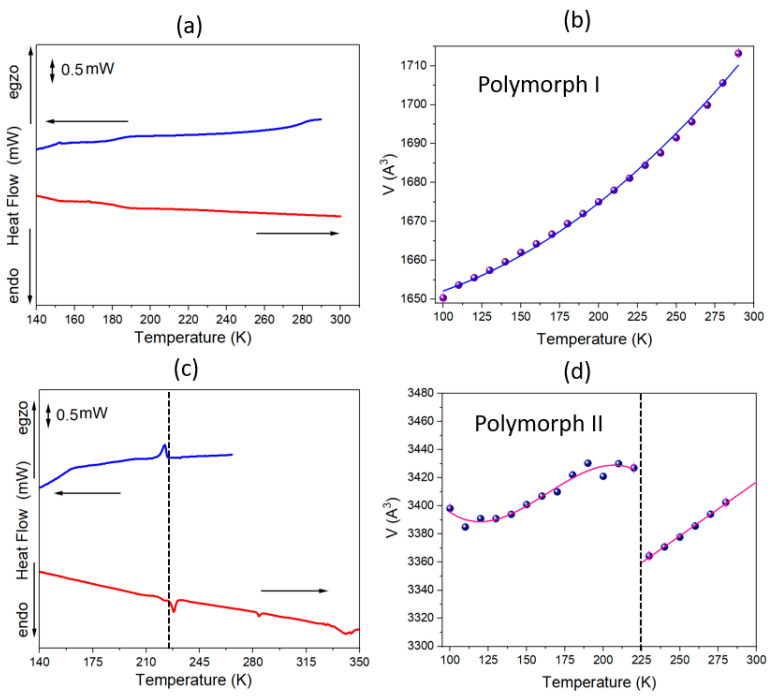
DCS curves (blue-cooling, red-heating) and the volume changes with cooling for polymorph **I** (**a**,**b**) and polymorph **II** (**c**,**d**). In (**b**,**d**) the lines are the guides for the eyes.

The single crystals of MHy_2_SbI_5_ crystallize in two polymorphic forms, orthorhombic **I** and monoclinic **II**. Polymorph **I** is stable and does not undergo temperature-induced phase transitions, while polymorph **II** undergoes a reversible phase transition, manifested by endothermic/exothermic peaks at 222 K/227 K during the cooling/heating runs on the differential scanning calorimetry curves (DSC). The phase transition is of a first-order character with a thermal hysteresis of 5 K and a discontinues volume change of approximately 60 Å^3^. Figure 1 depicts DSC runs along with the volume change measured during cooling for both polymorphs. Above 370 K, polymorph **II** undergoes a reconstructive phase transition, transforming into polymorph **I**. The low-temperature phase transition in polymorph **II** is also confirmed by an anomaly in the electric permittivity. Figure S1a,b in the Supplementary Information File (SI) displays the temperature dependence of the real ε′ (a) and imaginary ε″ (b) parts of the dielectric permittivity at various frequencies. The weak anomaly around 230 K (on heating) corresponds to the phase transition observed in the DSC. The abrupt changes in lattice parameters (Figure S1c,d) confirm a first order character of this transformation. 

### 2.2. Crystal Structure of MHy_2_SbI_5_

Both polymorphic forms have the same organic–inorganic one-dimensional (1D) perovskite structure built of [SbI_5_]^2−^_∞_ zig-zag chains of corner-sharing SbI_6_ octahedra. Polymorph **I** crystallizes in orthorhombic, acentric *P*2_1_2_1_2_1_ symmetry, as confirmed by the second-harmonic generation (SHG); refer to Figure S2 in SI. It may seem that this is another example of a structure in which the presence of MHy enforces an acentric arrangement of atoms. However, upon comparing the data on structures with the A_2_SbI_5_ stoichiometry it can be observed that the majority of them crystallize in non-centrosymmetric or polar space groups [26,51,52]. Hence, the absence of a center of symmetry in polymorph **I** should be attributed to the unique properties of this specific stoichiometry.

Polymorph **II** adopts a centrosymmetric, monoclinic structure with the *P*2_1_/*n* space group, and its unit cell is a 2**c**_ortho_ superstructure of polymorph **I**. Detailed information regarding the basic structural parameters of both crystals, data collection and refinement results obtained by single-crystal X-ray diffraction are presented in Table 1.

In polymorph **I,** the asymmetric unit consists of two disordered protonated methylhydrazinium cations and the SbI_5_^2−^ anion (Figure S3), which forms a one-dimensional zig-zag chain through corner-sharing SbI_6_^3−^ octahedra. In polymorph **II,** the asymmetric unit comprises four protonated MHy cations and two inorganic SbI_5_^2−^ anions (Figure S4). Two MHy cations are disordered over two positions, with occupancies of 0.75/0.25 and 0.62/0.38, while the third one is disordered over three positions. 

In organic–inorganic hybrids, the order–disorder processes of the molecular part are usually the main driving forces for the phase transitions and structural distortions of inorganic substructures. In MHy_2_SbI_5_, in **I**, the ordering of MHy with temperature lowering does not bring about any important changes in the skeleton. The volume of the unit cell experiences normal, positive thermal expansion; the distortion parameters for the octahedra (calculated in Vesta [54]) change only slightly. The bond length distortion is very similar, with 0.045 at 295 K and 0.042 at 100 K, whereas the angle variance increases slightly after cooling from 2.8 deg.^2^ to 4.4 deg.^2^. Both MHy cations are ordered at 100 K and interact through the N–H···N hydrogen bond with a N···N distance of 3.08(2) Å, and weak N–H···I hydrogen bonds with the iodine acceptors, with a minimum N···I distance of 3.51(2) Å. 

In contrast to polymorph **I**, polymorph **II** undergoes transformations during both cooling and heating. At low temperatures, around 225 K, a phase transition occurs, associated with a lowering of symmetry, manifested by a complex diffraction pattern characteristic of pseudo-merohedral twining. It likely involves a symmetry reduction to the triclinic system and thus, due to the overlap of diffraction peaks, we were unable to determine the crystal structure of this phase. Moreover, upon heating to a temperature of ~370 K, a reconstructive phase transition occurs in which polymorph **II** transforms into an orthorhombic non-centrosymmetric form with a reduced unit cell, characteristic of polymorph **I**. The single-crystal diffraction data collected at 365 K, after cooling the crystal from 370 K, and the SHG signal which appears at high temperatures after *P*2_1_/*n*→*P*2_1_2_1_2_1_ transition (shown S2 in SI) constitute direct evidence of this transformation. This intriguing behavior of **II** arises from the arrangement of [SbI_5_]^2−^_∞_ chains in the crystal structure. 

**Figure 2:**
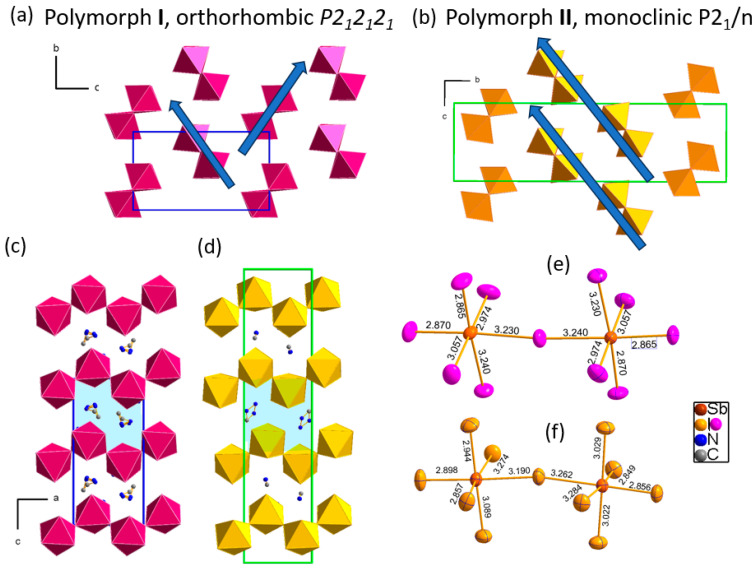
The crystal structure of orthorhombic polymorph **I** with acentric *P*2_1_2_1_2_1_ symmetry and monoclinic, centrosymmetric *P*2_1_*/n* polymorph **II**: (**a**,**b**) the packing of the inorganic part along *a*_ortho_, where herringbone and parallel motifs are highlighted by arrows; (**c**,**d**) [SbI_5_]^2−^_∞_ chains of corner-sharing octahedra in both **I** and **II**, with dissimilar structural motifs marked in blue; (**e**,**f**) the Sb–I bond lengths in both forms, with independent atoms drawn with front ellipsoids.

Both crystal structures essentially differ in the distribution of the 1D chains in the space. In the thermally stable polymorph **I**, the chains align in a herringbone-like configuration, ensuring optimal distances between neighboring chains. At room temperature, the shortest distances between chains are of 8.42 Å. In polymorph **II**, the arrangement of chains is different; there are two distinct alignments of chains, herringbone and nearly parallel. In the parallel configuration, the shortest distances between planes are equal to 7.74 Å, which stays at the origin of the structural instability of this form. Such a short distance between the chains is the shortest observed in the family of A_2_SbI_5_. Figure 2 illustrates the arrangements of the crystal structure in both polymorphic forms and Sb–I distances. 

The arrangement of chains in the structure and associated with the packing disorder of MHy cations seem to be the crucial differences between both polymorphic forms. The geometry of the chains in both **I** and **II** does not differ significantly from each other. Figures S5 and S6 in SI show the details of [SbI_5_]^2−^_∞_ chains for both polymorphs. The local symmetry of antimony ions is the lowest possible, C_1_, both in **I** and **II**. In **I** there is one inequivalent Sb(1) position, whereas in **II** there are two independent Sb(1) and Sb(2) atomic sites. The Sb-I distances at room temperature in **I** are distributed within 2.87–3.24 Å, whereas in **II** they are within 2.86–3.27 Å; the Sb–I–Sb angles between neighboring octahedra are equal to 174 deg. in **I**, whereas they range from 170 to 171 in **II**.

### 2.3. Luminescence Properties

The room temperature absorption spectra of both orthorhombic polymorph **I** and monoclinic polymorph **II** of 1D MHy_2_SbI_5_ cover the entire UV-Vis range up to 650 nm (Figure S7). Based on the reflectance spectrum, the energy band gap (*E_g_*) can be estimated using the Kubelka–Munk relation, also called remission function [55]:

(1) $$F\left(R\right)=\frac{{\left(1-R\right)}^{2}}{2R}$$

where *R* is reflectance. However, the modification proposed by Tauc allows the determination of *E_g_* through the graphical examination of the function [56]:

(2) $${\left[F(R\cdot hv)\right]}^{n}=B(hv-E_{g})$$

where h is the Planck constant, v denotes the photon’s frequency, and *B* is a constant. The value of the n  factor depends on the type of electron transition and equals 1/2 or 2 for direct and indirect transition band gaps, respectively [57,58] (Figures S8 and S9). Assuming that both polymorphs have a direct band gap, as indicated by many examples in the literature [38,59], the band gap values are comparable and equal 2.01 eV for orthorhombic polymorph **I** and 2.12 eV for the monoclinic polymorph **II**. The obtained values are also in good agreement with the *E_g_* of the low-dimensional hybrid halides based on Sb(III) [38,45,59,60,61]. 

**Figure 3:**
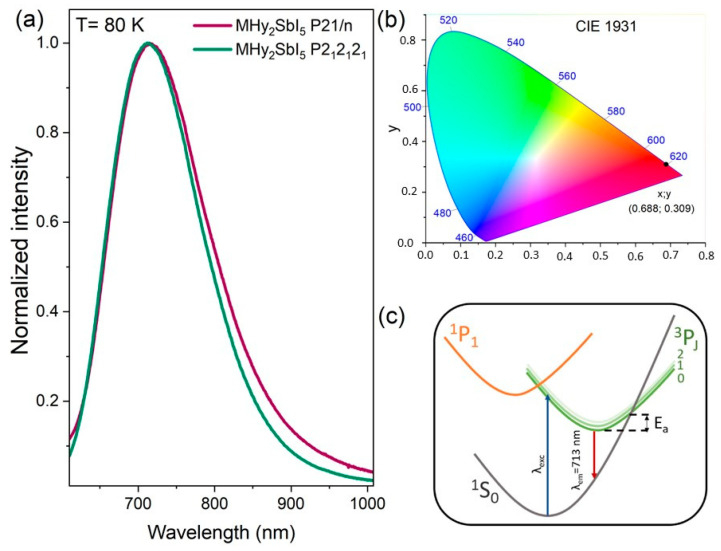
(**a**) Comparison of the emission spectra of MHy_2_SbI_5_ samples recorded at 80 K, (**b**) CIE diagram showing *x*, *y* coordinates, and (**c**) simple energy level diagram of Sb(III) ions.

The emission spectra recorded at 80 K are presented in Figure 3a. Both polymorphs exhibit broad emission spectra ranging from 625 nm to 1000 nm, associated with the active lone pair of 5s^2^ of Sb(III) ions (Figure 3c). Upon excitation by the 375 nm line, the electron is transferred to the ^3^P_2_ excited state of Sb(III) ions. Due to the small energy separation between ^3^P_J_ states, energy can be easily transferred to these levels. As a result, a broad luminescence band with FWHM of 0.34 eV and a large Stokes shift of around 1.57 eV is visible. Similar types of emission have been reported recently for other low-dimensional halides with antimony ions [38,45,59]. The shape as well as the position of the band maximum (713 nm) do not change with the crystal structure. Both MHy_2_SbI_5_ samples have red emission with x, y coordinates of 0.688 and 0.309 (Figure 3b). 

The lack of dependence of PL emission on the crystal structure confirms the slight differences in the antimony ion environment in both polymorphs described in the previous chapter. The octahedral distortion parameters collected in Table 2 show only minor changes in the coordination sphere of antimony in both polymorphs and their temperature-activated modifications. It is worth noting that direct antimony coordination is crucial from the perspective of energy transfer in these compounds. The activity of the 5s^2^ lone electron pair in the octahedral environment of antimony ions manifests in the off-center displacement of Sb ions from the symmetry center. In both compounds, the shifts are small, around 0.17 Å in **I** and 0.13 Å in **II** at room temperature. The large displacement parameters in both antimony and iodide positions suggest dynamic changes in the geometry around the antimony ion and thus dynamic changes of the stereo-activity of 5s^2^. With temperature lowering, the off-center displacement of Sb is smaller compared to room temperature (equal to 0.09 in **I** at 100K), which means that instability and dynamic changes associated with thermally activated atom vibrations impact the ability to effectively transfer energy, which, in turn, affects light emission in the PL process.

**Figure 4:**
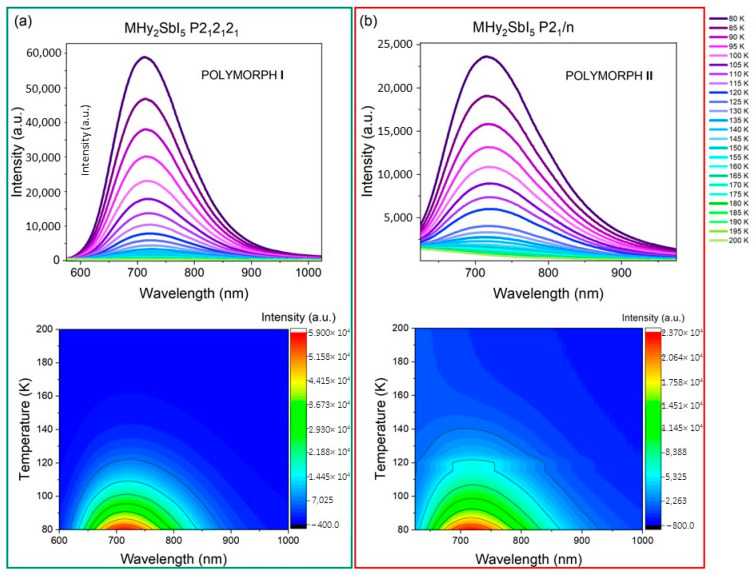
Temperature-dependent emission spectra and intensity contour maps of (**a**) MHy_2_SbI_5_, *P*2_1_2_1_2_1_, polymorph **I** and (**b**) MHy_2_SbI_5_, *P*2_1_/*n*, polymorph **II**.

The temperature-dependent emission spectra of investigated samples were recorded every 5 K (Figure 4). As shown, the intensity of the emission significantly decreases with temperature, but the shape, as well as the band position, do not change (Figure S10). The emission is not very stable, and the temperature quenching T_0.5_ is 96 K and 99 K for MHy_2_SbI_5_ polymorphs **I** and **II**, respectively. The energy activation for thermal quenching calculated using the Boltzmann function equals 79 meV and 60 meV for polymorphs **I** and **II**, respectively (Figures S11 and S12). 

## 3. Conclusions

In this study, we investigate the properties of a new 1D organic–inorganic hybrid compound, MHy_2_SbI_5_, with a focus on its crystal structure and luminescent behavior. This hybrid material crystallizes in two polymorphic forms (**I** and **II**), exhibiting distinct thermal and structural characteristics. Polymorph **I** adopts an acentric *P*2_1_2_1_2_1_ chiral space group and remains stable without temperature-induced phase transitions. In contrast, polymorph **II** undergoes a reversible low-temperature phase transition and a high-temperature reconstructive transformation to polymorph **I**. Both crystal structures consist of 1D perovskite zig-zag chains of corner-sharing SbI_6_ octahedra. The structural differences between them are attributed to the spatial arrangement of [SbI_5_]^2−^_∞_ chains in the crystal structure.

The luminescent properties of both polymorphs are characterized by a red photoluminescence (PL) originating from the 5s^2^ electron pairs of Sb(III) ions. The emission spectra recorded at 80 K show broad emission bands ranging from 625 nm to 1000 nm. The energy band gap (E_g_) values, estimated based on the UV-Vis absorption spectra, indicate that both polymorphs have direct band gaps, with E_g_ values of 2.01 eV and 2.12 eV for polymorph **I** and **II**, respectively. Interestingly, the lack of dependence of PL emission on the crystal structure suggests only slight differences in the antimony(III) ion environment in both polymorphs. The octahedral distortion parameters show minor changes in the coordination sphere of antimony(III) in both polymorphs and their temperature-activated modifications.

In summary, the study provides insights into the structural and luminescent characteristics of the new organic–inorganic MHy_2_SbI_5_ with a 1D polymeric arrangement of antimony iodide complex ions, filling a gap in PL research in this class of materials and contributing to the understanding of structure-property relations in lead-free 1D perovskites.

## 4. Experimental Details 

### 4.1. Synthesis

All materials needed for the synthesis of [NH_2_-NH_2_-CH_3_]_2_SbI_5_ (MHIA-I) and [NH_2_-NH_2_-CH_3_]_2_SbI_5_ (MHIA-II) were purchased from commercial sources (Sigma-Aldrich and Merck (HI)) and used without further purification: [NH_2_-NH_2_-CH_3_] (98%), SbI3 (>99.998%), HI (57%). The crystals were grown by a slow evaporation of a concentrated HI solution containing the 2:1 ratio of [NH_2_-NH_2_-CH_3_] and SbI_3_. The salts obtained were twice recrystallized from a methanol solution and their composition was verified by an elemental analysis: MHIA-I (C: 2.80 (theor. 2.82%), N: 6.52% (theor. 6.59%), H: 1.72% (theor. 1.66%) and MHIA-II (C: 2.73 (theor. 2.82%), N: 6.55% (theor. 6.59%), H: 1.70% (theor. 1.66%). The single crystals suitable for X-ray measurements were grown from an aqueous solution at a constant room temperature. Interestingly, modifications I and II of the MHy_2_SbI_5_ derivative were found to crystallize simultaneously from the methanol solution. Both polymorphs are dark red (burgundy) in color and crystallize in the form of well-shaped rhombohedrons. In addition to single-crystal X-ray diffraction measurements, absorption and emission measurements were conducted on single crystals verified by diffraction and subsequently powdered. A similar methodology was chosen for DSC and BDS measurements; however, due to the larger amount of material needed for the experiment, the possibility of mixing both phases must be considered.

### 4.2. Single-Crystal X-Ray Diffraction

Single-crystal X-ray diffraction measurements were obtained using the Oxford Diffraction Xcalibur four-circle diffractometer, which operates with an Atlas CCD detector and graphite-monochromated Mo Kα radiation. The absorption correction was performed using the multiscan method in CrysAlis PRO 1.171.39.46 (Rigaku Oxford Diffraction, 2018). The solution and refinement of the crystal structure were carried out in Olex2 1.5 [62]. SHELXT [63]; SHELXL [64]. The diffraction from two polymorphic forms was collected at 100 K, 295 K (orthorhombic, polymorph **I**) and 290 K, 365 K for monoclinic, polymorph **II**. All structures were deposited in the CCDC database, with 2322356, 2322357, 2322359 and 2322360 accession numbers. The detailed experimental and geometric parameters are given in Table S1 and S2 in the Supplementary Information File (SI). 

The single-crystal structures of polymorph **I** were determined at 295 K and 100 K, revealing that it belongs to an orthorhombic system with a chiral space group *P*2_1_2_1_2_1_. The crystal structure of polymorph **I** was refined as a two-component inversion twin, with a refined twin component ratio of 0.6:0.4 at 295 K and 100 K. At 295 K, two crystallographically independent MHy cations are observed, both exhibiting disorder. The first is disordered in two positions with an occupancy ratio of 0.7:0.3, while the second one is disordered in two equivalent positions (0.5:0.5 occupancy ratio) and refined isotropically. While no phase transition was observed in polymorph **I**, cooling the crystal to 100 K revealed alterations in the organic part of the crystal structure. Both Mhy cations order and occupy one position in the structure with full occupancy. During the structural refinement of polymorph **I**, SHELXL restraint instructions (SADI, SIMU, DFIX) were used to manage disordered moieties.

Polymorph **II** crystallizes in the centrosymmetric monoclinic *P*2_1_/*n* space group. In the room-temperature phase, all four symmetry-independent MHy cations exhibit disorder, with two of them being disordered over two positions, having occupancies of 0.75(2) and 0.25(2), and 0.62(3) and 0.38(3), respectively. SADI restraints were employed to appropriately model the disordered cationic moiety. The atoms of the minor orientation were refined isotropically. The third and fourth organic moieties were refined only isotropically, and the disorder in the latter cation was modeled using a three-site model. In the high-temperature phase of polymorph **II**, two independent cations were disordered within equivalent positions. Refinement was conducted with SADI, SIMU and DFIX restraint to control disordered moieties. Due to the heavy twinning of polymorph **II** below 220 K, the single-crystal X-ray diffraction for the low-temperature phase experiment did not yield sufficient data for structure solution.

### 4.3. Thermal Analysis 

DSC measurements were conducted using a Metler Toledo DSC 3 instrument. Polycrystalline samples were cooled and heated in the range of 120 K–360 K; ramp rate was 10 K/min. 

### 4.4. Electrical Measurements

Electrical measurements were performed for polycrystalline samples in the temperature range of 180 K–300 K. The samples were painted with silver-conductive paint. Agilent E4980A Precision LCR Meter in the frequency range of 135 Hz–2 MHz was used. Temperature was stabilized and controlled by an INSTEC STC200. The procedures were carried out in a controlled nitrogen atmosphere. 

### 4.5. Optical Measurements

The room temperature absorption spectra of the powdered samples were measured by using a Varian Cary 5E UV–Vis–NIR spectrophotometer (Varian, Palo Alto, CA, USA). Emission spectra at different temperatures under 375 nm excitation from a laser diode were measured with the Hamamatsu photonic multichannel analyzer PMA-12, equipped with a BT-CCD linear image sensor (Hamamatsu Photonics, Iwata, Japan). The temperature of the samples was controlled by using a Linkam THMS 600 Heating/Freezing Stage (Linkam, Tadworth, UK).

## Figures and Tables

**Table 1:** Crystal data, collection and refinement results for MHy_2_SbI_5_.

Crystal Data	Polymorph I		Polymorph II	
Chemical Formula	(CN_2_H_7_)_2_[SbI_5_]			
Molecular weight (Formula mass/g·mol^−1^) Crystal system Space group	836.31	836.31	836.31	836.31
	Orthorhombic	Orthorhombic	Monoclinic	Orthorhombic
	*P*2_1_2_1_2_1_	*P*2_1_2_1_2_1_	*P*2_1_*/n*	*P*2_1_2_1_2_1_
Temperature (K)	100	295	290	365
*a* [Å]	8.901 (3)	8.835 (3)	8.648 (3)	8.737 (3)
*b* [Å]	10.291 (4)	10.550 (4)	37.271 (9)	10.689 (4)
*c* [Å]	18.004 (5)	18.395 (5)	10.668 (4)	18.754 (5)
*V* [Å^3^]	1649.2 (10)	1714.6 (10)	3437.7 (19)	1751.4 (10)
β [◦]			91.32 (3)	
*Z*	4	4	8	4
Data collection				
No. of measured, independent, observed [I > 2σ(I)] reflections	12,731, 3129, 3018	15,315, 3244, 2913	57,907, 8809, 5784	15,653, 3325, 1711
*R*_int_	0.027	0.025	0.035	0.057
Refinement				
R[*F*^2^ > 2σ(*F*^2^)],	0.032	0.028	0.035	0.048
wR(*F*^2^), S	0.079, 1.08	0.064, 1.05	0.087, 1.05	0.157, 1.02
Δρmax, Δρmin (e Å^−3^)	2.39, −1.52	0.82, −0.77	0.99, −0.86	0.92, −0.85
Twin refinement	Refined as an inversion twin			
Absolute structure parameter	0.38 (14)	0.43 (13)		0.34 (12)

**Table 2:** Octahedral distortion parameters in both **I** and **II** calculated in Vesta [54].

	Polymorph I		Polymorph II	
Temperature	100 K	295 K	290 K	365 K
Average bond length (Å)	3.024	3.039	3.041	3.050
Polyhedral volume (Å^3^)	36.81	37.39	37.42	37.75
Distortion index (bond length)	0.041	0.045	0.047	0.052
Bond angle variance (deg.^2^)	4.38	2.78	6.61	6.41

## Data Availability

Experimental data will be available at https://doi.org/10.5281/zenodo.10471676 accessed on 16 January 2024. Crystal structures have been deposited in CCDC with numbers 2249176, 2249177 and 2249178.

## Supplementary Materials

The following supporting information can be downloaded at: https://www.mdpi.com/article/10.3390/molecules29020455/s1, Figure S1: Temperature dependence of the real ε′ (**a**) and imaginary ε″ part (**b**) of the complex electric permittivity measured for the polymorph **II** in heating cycle; (**c**,**d**) thermal evolution of lattice parameters in polymorph **II** with cooling; Figure S2: (**a**) Second harmonic generation (SHG)—temperature dependence for Polymorph **I**; (**b**) SHG signal appears after the reconstructive phase transition from centrosymmetric polymorph **II** to non-centrosymmetric polymorph **I**, (**c**) the SHG signal from polymorph **I** at room temperature in relation to KDP. Figure S3: The independent parts of Polymorph **I** in 100 K (**a**) and 295 K (**b**). Displacement ellipsoids are drawn at the 50% and 30% probability levels for 100 K and 295 K, respectively. [Symmetry codes: (i) 1/2 + x, 3/2 − y, 1 − z]; Figure S4. The independent part of polymorph **II** is shown in 290 K (**a**) in the monoclinic phase *P*2_1_/*n* and at 365 K (**b**) in the orthorhombic phase *P*2_1_2_1_2_1_. Displacement ellipsoids are drawn at the 30% probability levels. [Symmetry codes: (*i*, a) −1 + *x*, *y*, *z*; (*i*, b); −1/2 + *x*, 1/2 − *y*, 1 − *z*]; Figure S5. A view of the inorganic chains in the crystal structure of polymorph **I** along *a*-axis in 295 K (**a**) and 100 K (**b**); Figure S6. A view of the inorganic chains in the crystal structure of polymorph **II** along *a*-axis in 295 K (**a**) and 365 K (**b**); Figure S7. Diffuse reflectance spectra of investigated hybrid 1D MHy_2_SbI_5_ perovskites; Figure S8**.** Energy band gap of the MHy_2_SbI_5_ *P*2_1_/*n* crystals; Figure S9. Energy band gap of the MHy_2_SbI_5_ *P*2_1_2_1_2_1_ crystals; Figure S10**.** Integrated intensity in function of temperature of the investigated hybrid 1D MHy_2_SbI_5_ perovskites; Figure S11. Energy activation for the thermal quenching of the MHy_2_SbI_5_ *P*2_1_2_1_2_1_ crystals; Figure S12. Energy activation for the thermal quenching of the MHy_2_SbI_5_ *P*2_1_/*n* crystals; Table S1: Selected geometric parameters (Å, º) for polymorph **I**; Table S2. Selected geometric parameters (Å, º) for polymorph **II**. Table S3. Crystal data, collection and refinement results for (MHy)_2_[SbI_5_].
