# Supplementary material for: Polymorphism and Red Photoluminescence Emission from 5s2 Electron Pairs of Sb(III) in a New One-Dimensional Organic–Inorganic Hybrid Based on Methylhydrazine: MHy2SbI5
Source: Molecules. 2024 Jan 17;29(2):455. doi: 10.3390/molecules29020455 (PMC10821241; doi:10.3390/molecules29020455)
Supplement: Supplementary file 1 [file molecules-29-00455-s001.zip › molecules-2825156-supplementary.pdf]

Supplementary Information

Polymorphism and Red Photoluminescence Emission from 5s2 Electron Pairs of Sb(III) in a New One-Dimensional Organic–Inorganic Hybrid Based on Methylhydrazine: MHy2SbI5

Magdalena Rowińska [1], Dagmara Stefańska [1], Tamara J. Bednarchuk [1], Jan K. Zaręba [2], Ryszard Jakubas [3] and Anna Gągor [1,*]

[1] Institute of Low Temperature and Structure Research, Polish Academy of Sciences, Okólna 2, 50-422 Wrocław, Poland

[2] Advanced Materials Engineering and Modelling Group, Wrocław University of Science and Technology, Wyb. Wyspiańskiego 27, 50-370 Wrocław, Poland

[3] Faculty of Chemistry, University of Wrocław, F. Joliot-Curie 14, 50-383 Wrocław, Poland

[*] Correspondence: a.gagor@intibs.pl

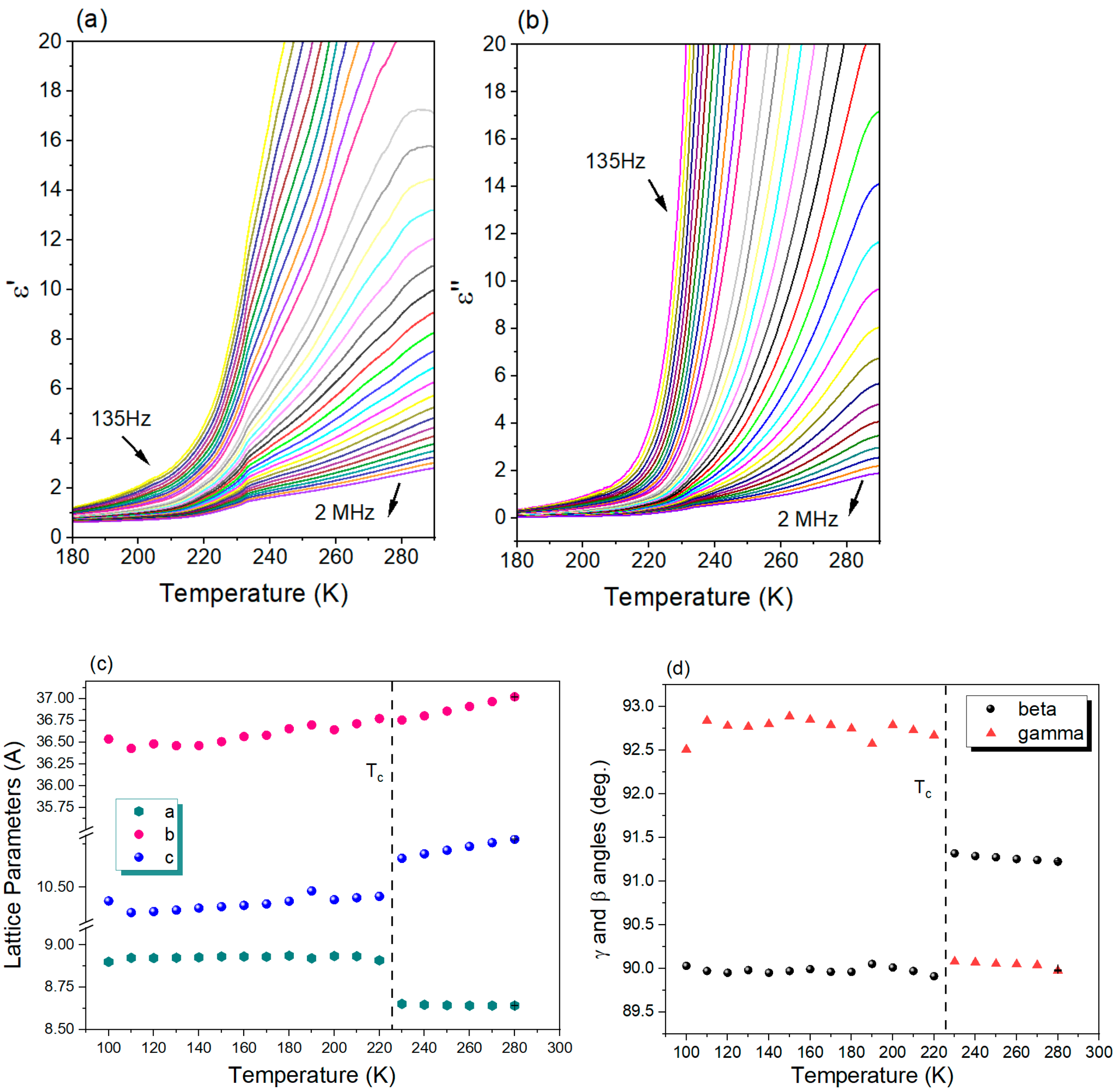

Figure S1. Temperature dependence of the real ε' (a) and imaginary ε'' part (b) of the complex electric permittivity measured for the Polymorph **II** in heating cycle. Dielectric anomaly associated with the phase transition is small, overshadowed by the conductivity; (c-d) thermal evolution of lattice parameters in polymorph **II** with cooling. An abrupt changes of the lattice parameters and monoclinic angle are observed at the low-temperature phase transition.

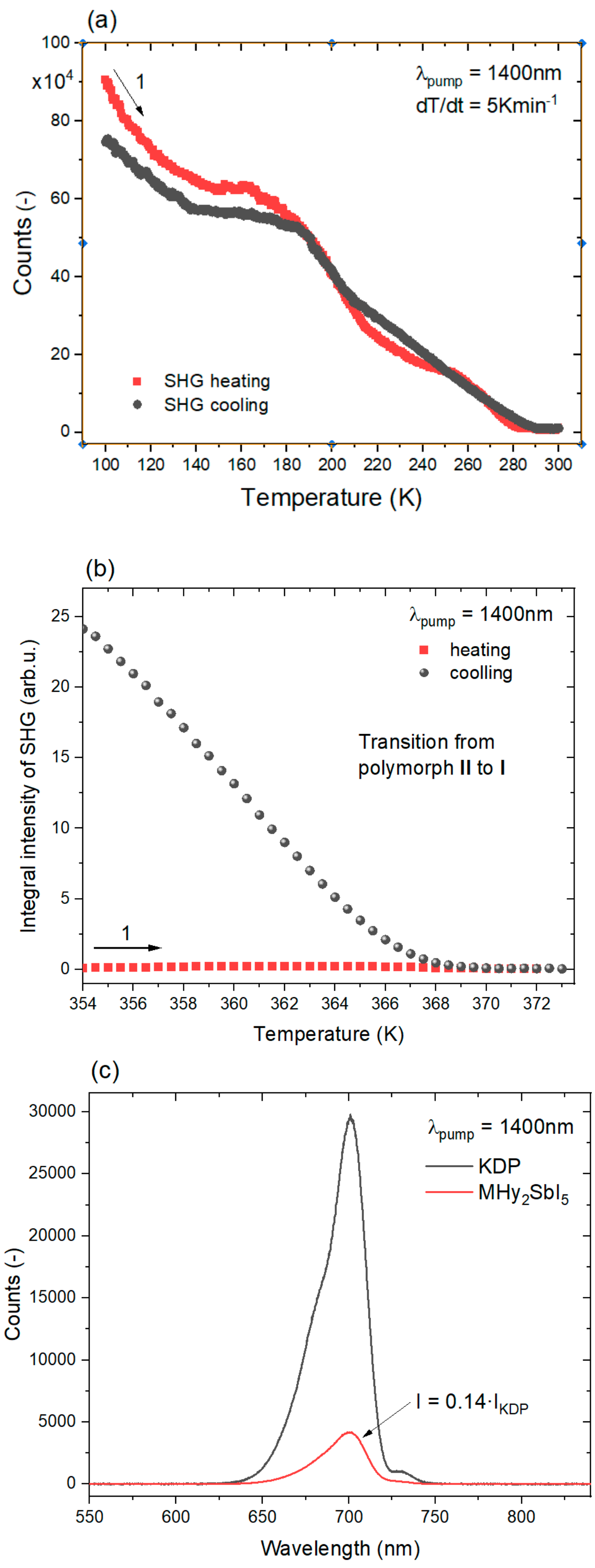

Figure S2. (a) Second harmonic generation (SHG) - temperature dependence for Polymorph **I,** (b) SHG signal appears after the reconstructive phase transition from centrosymmetric polymorph **II** to non-centro symmetric polymorph **I**, (c) the SHG signal from polymorph **I** at room temperature in relation to KDP.

Table S1. Selected geometric parameters (Å, º) for Polymorph **I**.

**Polymorph I T=295K**		**Polymorph I T=100K**	
I1—Sb1	2.9740 (13)	I1—Sb1	2.9939 (14)
I2—Sb1	2.8699 (12)	I2—Sb1	2.8563 (14)
I3—Sb1	2.8647 (12)	I3—Sb1	2.8675 (14)
I4—Sb1	3.0570 (12)	I4—Sb1	3.0056 (14)
I5—Sb1^{i}	3.2399 (12)	I5—Sb1^{i}	3.2001 (13)
I5—Sb1	3.2298 (12)	I5—Sb1	3.2229 (14)
Sb1—I5—Sb1^{i}	173.62 (3)	Sb1^{i}—I5—Sb1	169.48 (3)
I1—Sb1—I4	179.50 (4)	I1—Sb1—I4	177.67 (4)
I1—Sb1—I5^{ii}	89.65 (3)	I1—Sb1—I5	93.20 (4)
I1—Sb1—I5	90.49 (4)	I1—Sb1—I5^{ii}	89.06 (4)
I2—Sb1—I1	89.28 (4)	I2—Sb1—I1	88.55 (4)
I2—Sb1—I4	90.92 (4)	I2—Sb1—I3	93.17 (4)
I2—Sb1—I5	175.59 (4)	I2—Sb1—I4	90.62 (4)
I2—Sb1—I5^{ii}	89.13 (4)	I2—Sb1—I5^{ii}	89.74 (4)
I3—Sb1—I1	89.44 (4)	I2—Sb1—I5	177.60 (4)
I3—Sb1—I2	93.71 (4)	I3—Sb1—I1	87.65 (4)
I3—Sb1—I4	90.10 (4)	I3—Sb1—I4	90.22 (4)
I3—Sb1—I5	90.69 (4)	I3—Sb1—I5^{ii}	175.55 (4)
I3—Sb1—I5^{ii}	177.01 (4)	I3—Sb1—I5	88.54 (4)
I4—Sb1—I5^{ii}	90.80 (3)	I4—Sb1—I5^{ii}	93.11 (4)
I4—Sb1—I5	89.35 (3)	I4—Sb1—I5	87.69 (4)
I5—Sb1—I5^{ii}	86.47 (3)	I5^{ii}—Sb1—I5	88.65 (3)

Symmetry code(s): (*i*) *x*-1/2, -*y*+3/2, -*z*+1; (*ii*) *x*+1/2, -*y*+3/2, -*z*+1.

Table S2. Selected geometric parameters (Å, º) for Polymorph **II**.

**Polymorph II T=290K**		**Polymorph II T=365K**	
Sb1—I2	2.8563 (11)	Sb1—I1^{i}	3.288 (2)
Sb1—I5	3.2616 (12)	Sb1—I1	3.276 (3)
Sb1—I1	2.8491 (11)	Sb1—I3	3.061 (2)
Sb1—I4	3.0217 (9)	Sb1—I2	2.849 (2)
Sb1—I3	3.0292 (9)	Sb1—I4	2.863 (3)
Sb2—I5	3.1902 (10)	Sb1—I5	2.968 (3)
Sb2—I6	3.2740 (11)	I1—Sb1—I1^{i}	83.51 (4)
Sb2—I9	2.8565 (10)	I3—Sb1—I1^{i}	90.81 (7)
Sb2—I8	3.0886 (9)	I3—Sb1—I1	90.45 (7)
Sb2—I10	2.8982 (10)	I2—Sb1—I1	91.39 (8)

Sb2—I7	2.9437 (9)	I2—Sb1—I1^{i}	174.83 (9)
I2—Sb1—I5	175.25 (2)	I2—Sb1—I3	90.09 (8)
I2—Sb1—I4	90.84 (3)	I2—Sb1—I4	94.91 (8)
I2—Sb1—I3	90.45 (3)	I2—Sb1—I5	89.12 (9)
I1—Sb1—I2	94.91 (3)	I4—Sb1—I1	173.64 (8)
I1—Sb1—I5	89.69 (3)	I4—Sb1—I1^{i}	90.18 (8)
I1—Sb1—I4	88.64 (3)	I4—Sb1—I3	90.39 (8)
I1—Sb1—I3	90.06 (3)	I4—Sb1—I5	89.21 (8)
I4—Sb1—I5	90.44 (3)	I5—Sb1—I1^{i}	90.02 (8)
I4—Sb1—I3	178.24 (2)	I5—Sb1—I1	90.04 (8)
I3—Sb1—I5	88.37 (3)	I5—Sb1—I3	179.08 (8)
I5—Sb2—I6	83.19 (3)		
I9—Sb2—I5	91.52 (3)		
I9—Sb2—I6	174.69 (2)		
I9—Sb2—I8	90.10 (3)		
I9—Sb2—I10	94.67 (3)		
I9—Sb2—I7	90.39 (3)		
I8—Sb2—I5	89.10 (3)		
I8—Sb2—I6	90.16 (3)		
I10—Sb2—I5	173.79 (2)		
I10—Sb2—I6	90.63 (3)		
I10—Sb2—I8	90.35 (3)		
I10—Sb2—I7	90.41 (3)		
I7—Sb2—I5	90.08 (3)		
I7—Sb2—I6	89.28 (3)		
I7—Sb2—I8	179.06 (2)		
Sb2—I5—Sb1	169.64 (2)		

Symmetry code(s): (*i*) *x*-1/2, -*y*+1/2, -*z*+1.

Table S3. Crystal data, collection and refinement results for MHy_2SbI_5.

Crystal data	**Polymorph I**		**Polymorph II**	
Chemical formula	$(CN_2H_7)_2[SbI_5]$			
Molecular weight M_r	836.31	836.31	836.31	836.31
Crystal system	Orthorhombic	Orthorhombic	Monoclinic	Orthorhombic
Space group	$P2_12_12_1$	$P2_12_12_1$	$P2_1/n$	$P2_12_12_1$
Temperature (K)	100	295	290	365
a [Å]	8.901 (3)	8.835 (3)	8.648 (3)	8.737 (3)
b [Å]	10.291 (4)	10.550 (4)	37.271 (9)	10.689 (4)
c [Å]	18.004 (5)	18.395 (5)	10.668 (4)	18.754 (5)
V [Å^3]	1649.2 (10)	1714.6 (10)	3437.7 (19)	1751.4 (10)
β [°]			91.32 (3)	
Z	4	4	8	4

Data collection				
No. of measured, independent, observed [I > 2s(I)] reflections	12731, 3129, 3018	15315, 3244, 2913	57907, 8809, 5784	15653, 3325, 1711
R_{int}	0.027	0.025	0.035	0.057
Refinement				
$R[F^2 > 2\sigma(F^2)]$, $wR(F^2)$, S	0.032, 0.079, 1.08	0.028, 0.064, 1.05	0.035, 0.087, 1.05	0.048, 0.157, 1.02
$\Delta\rho_{max}$, $\Delta\rho_{min}$ (e Å^{-3})	2.39, -1.52	0.82, -0.77	0.99, -0.86	0.92, -0.85
Twin refinement	Refined as an inversion twin			
Absolute structure parameter	0.38 (14)	0.43 (13)		0.34 (12)

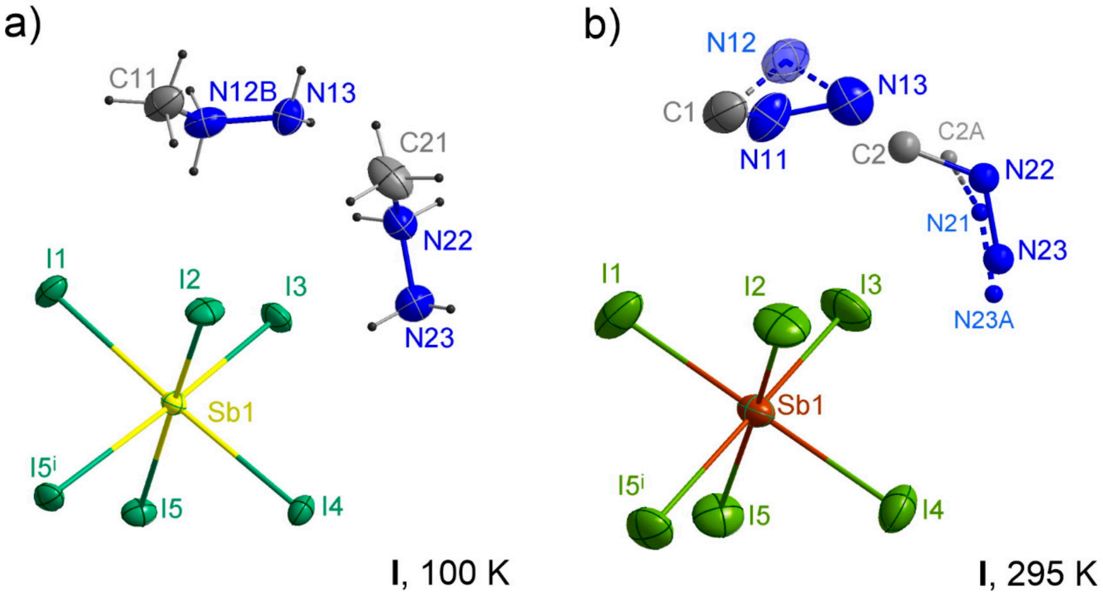

Figure S3. The independent parts of polymorph **I** in 100 K (a) and 295 K (b). Displacement ellipsoids are drawn at the 50% and 30% probability levels for 100 K and 295 K, respectively. [Symmetry codes: (*i*) 1/2+x, 3/2-y, 1-z].

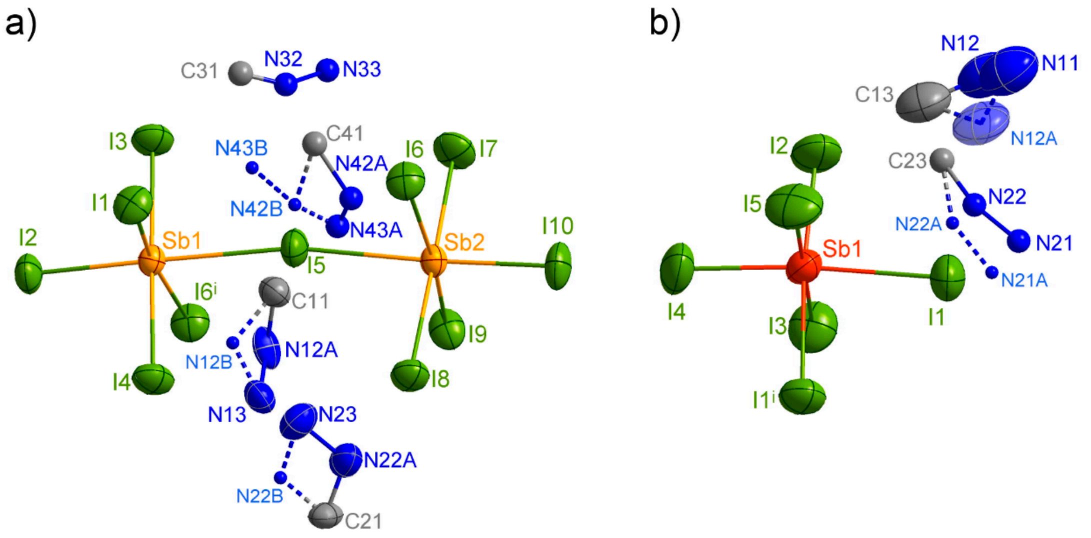

Figure S4. The independent part of polymorph **II** is shown in 290 K (a) in the monoclinic phase $P2_1/n$ and at 365 K (b) in the orthorhombic phase $P2_12_12_1$. Displacement ellipsoids are drawn at the 30 % probability levels. [Symmetry codes: (*i*, a) -1+*x*, *y*, *z*; (*i*, b); -1/2+*x*, 1/2-*y*, 1-*z*].

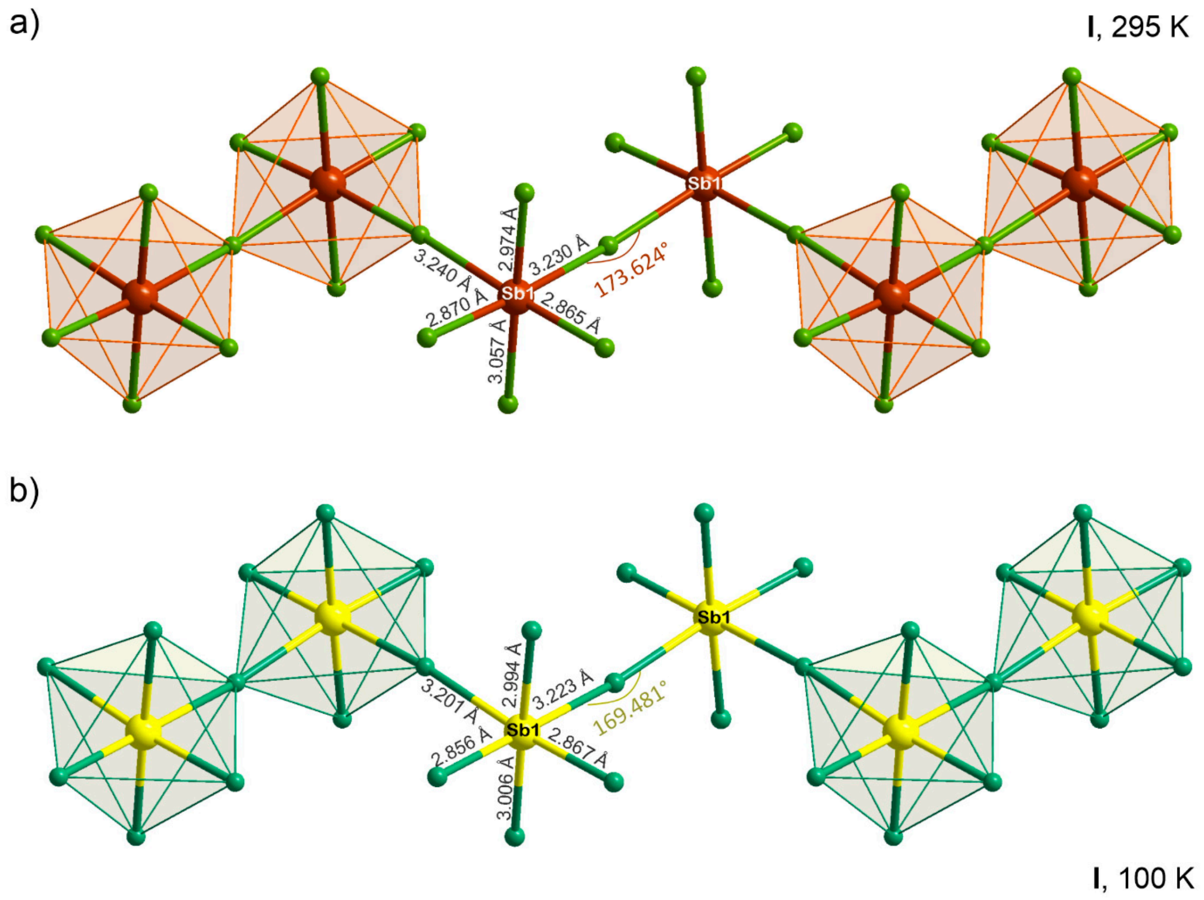

Figure S5. A view of the inorganic chains in the crystal structure of polymorph **I** along *a*-axis in 295 K (a) and 100 K (b).

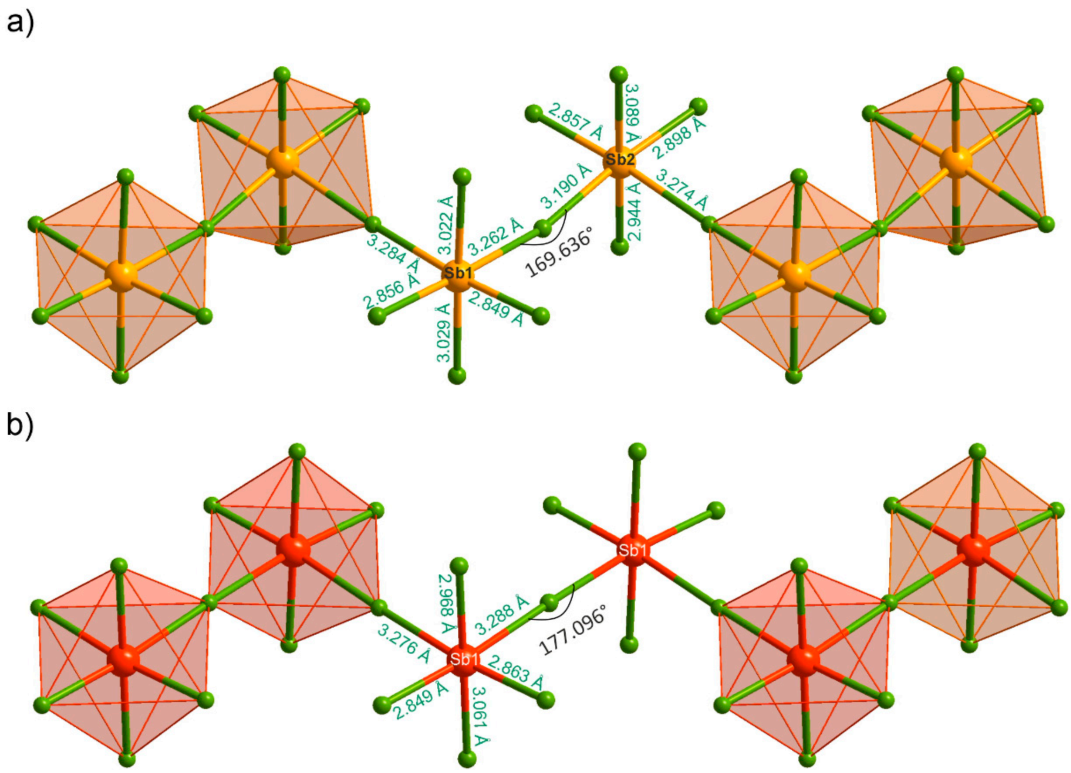

Figure S6. A view of the inorganic chains in the crystal structure of polymorph **II** along *a*-axis in 295 K (a) and 365 K (b).

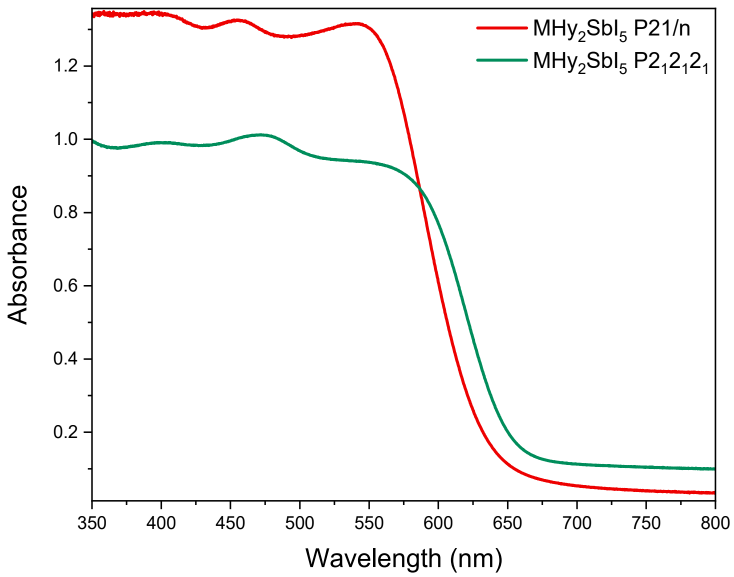

Figure S7. Diffuse reflectance spectra of investigated hybrid 1D MHy_2SbI_5 perovskites.

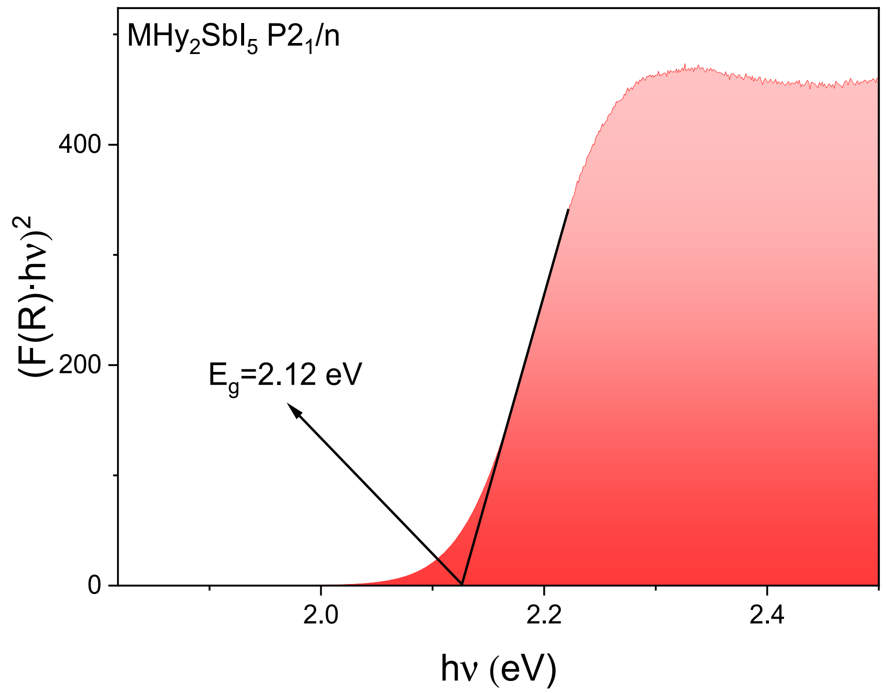

Figure S8. Energy band gap of the MHy_2SbI_5 $P2_1/n$ crystals, polymorph **II**.

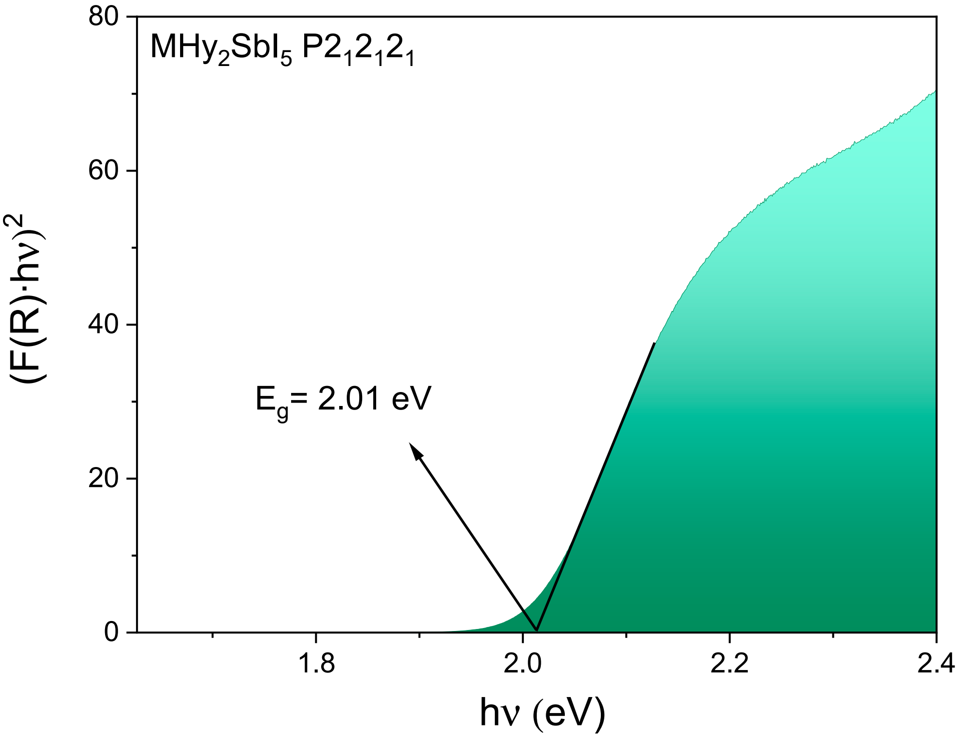

Figure S7. Energy band gap of the MHy_2SbI_5 $P2_12_12_1$ crystals, polymorph **I**.

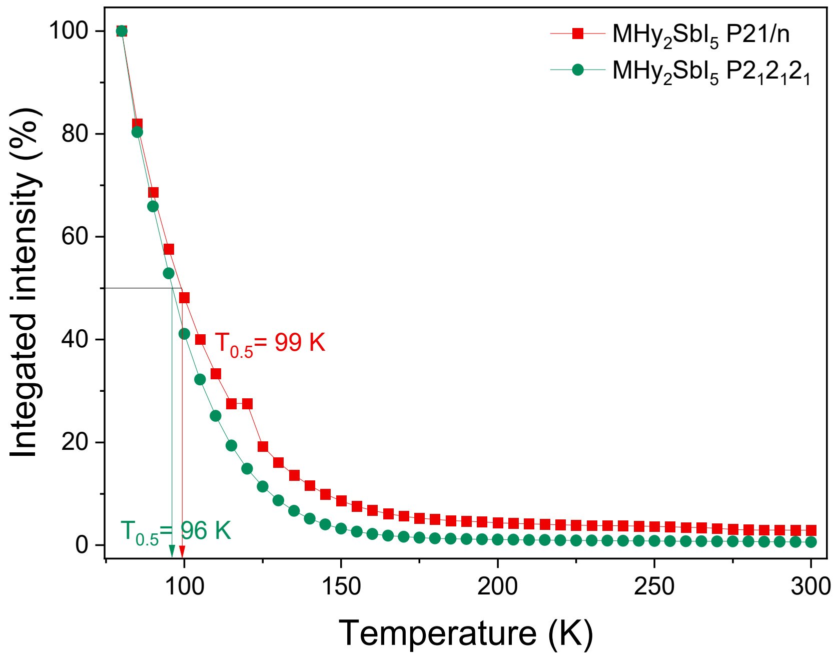

Figure S8. Integrated intensity in function of temperature of the investigated hybrid 1D MHy_2SbI_5 perovskites.

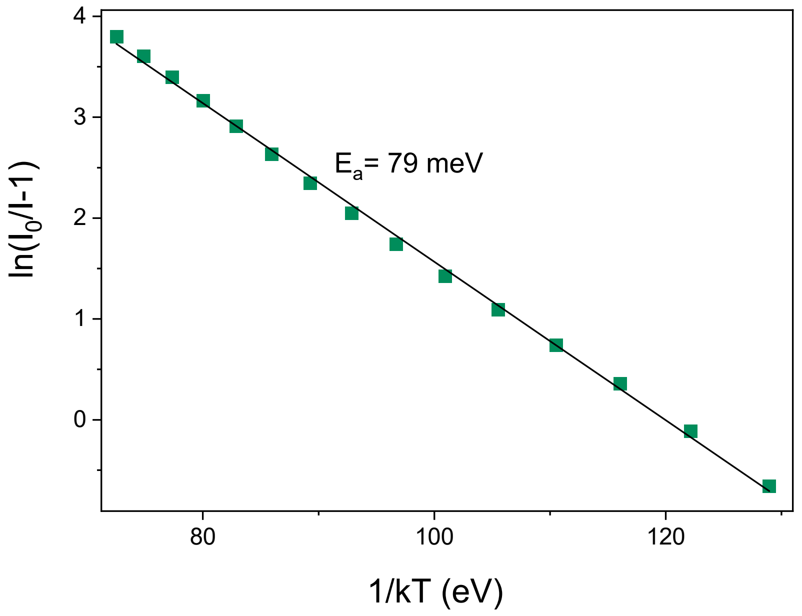

Figure S9. Energy activation for the thermal quenching of the MHy_2SbI_5 $P2_12_12_1$ crystals, polymorph **I**.

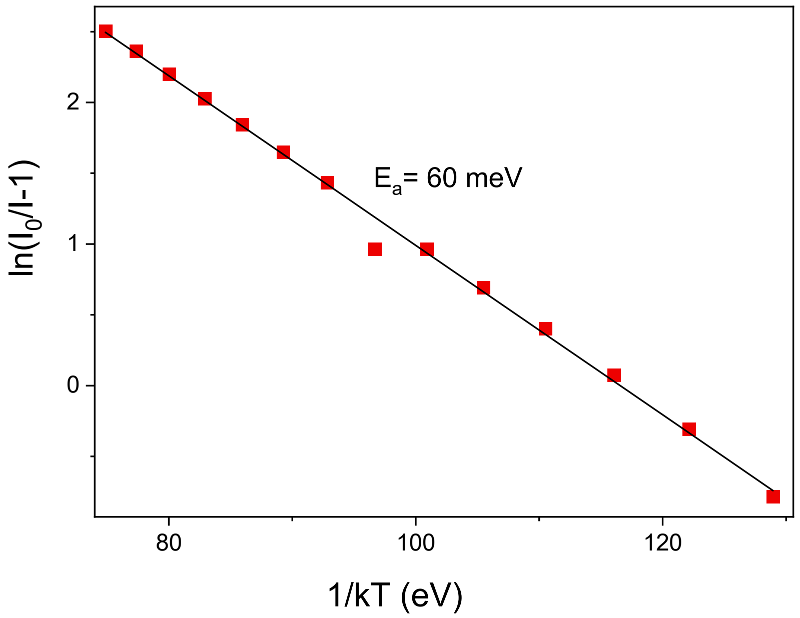

Figure S10. Energy activation for the thermal quenching of the MHy_2SbI_5 $P2_1/n$ crystals, polymorph **II.**
